# Adiponectin Signaling Dysfunction and Pro-inflammation in Perivascular Adipose Tissue in Non-obstructive Ischemic Heart Disease: A Narrative Literature Review of New Endocrine-Metabolic Therapeutic Targets

**DOI:** 10.7759/cureus.111759

**Published:** 2026-06-29

**Authors:** Paulina Elizabeth Cisneros Clavijo, Diego Fernando Pinzón Tejada, John Manuel Dorado Ramírez, Sergio Daniel Zabaleta Orozco, Nataisha Michaella Seidlitz Arteaga, Adolfo Martínez Hernández, Diego Adolfo Vimos Congacha, Rodolfo Granados Villa

**Affiliations:** 1 Endovascular Surgery, Hospital General Enrique Garcés, Quito, ECU; 2 Hemodynamics and General and Interventional Cardioangiology, Pontificia Universidad Catolica del Ecuador, Quito, ECU; 3 Emergency, Clinica Fundación Oftalmológica de Santander (FOSCAL), Bucaramanga, COL; 4 Internal Medicine, Instituto Mexicano del Seguro Social, Mexico City, MEX; 5 Medicine, Universidad Surcolombiana, Neiva, COL; 6 Medicine, Universidad Anáhuac Cancún, Cancún, MEX; 7 Internal Medicine, Instituto Mexicano del Seguro Social, Torreon, MEX; 8 Nephrology, Clínica de los Riñones MENYDIAL, Riobamba, ECU; 9 Medicine, Universidad de la Salud del Estado de Puebla (USEP), Puebla, MEX

**Keywords:** adiponectin, adipor1/adipor2, coronary microvascular dysfunction, endothelial dysfunction, inoca, minoca, perivascular adipose tissue

## Abstract

Ischemia with non-obstructive coronary arteries (INOCA)/myocardial infarction with non-obstructive coronary arteries (MINOCA) affects a substantial proportion of patients undergoing coronary angiography; however, its pathophysiology remains incompletely understood, and evidence-based management remains limited. This narrative review synthesizes mechanistic, translational, and clinical evidence linking pericoronary perivascular adipose tissue (PVAT) dysfunction to coronary microvascular dysfunction (CMD) and vasomotor dysregulation in the absence of epicardial stenosis. A comprehensive literature search was conducted across PubMed/MEDLINE, Scopus, the Cochrane Library, and Google Scholar up to March 2025, encompassing preclinical studies, observational cohorts, imaging studies, and clinical trials across five thematic domains: PVAT physiology, adiponectin signaling, PVAT phenotypic transition in INOCA/MINOCA, pericoronary fat attenuation index (FAI), and endocrine-metabolic therapeutic targets. In healthy individuals, PVAT maintains coronary homeostasis through adiponectin-mediated AMP-activated protein kinase (AMPK)-endothelial nitric oxide synthase (eNOS)-nitric oxide signaling and anti-inflammatory paracrine effects. Under metabolic dysfunction, PVAT undergoes a phenotypic transition characterized by reduced adiponectin secretion and increased production of tumor necrosis factor-α (TNF-α), interleukin-6 (IL-6), and reactive oxygen species, thereby impairing endothelium-dependent vasodilation and promoting microvascular dysfunction. In MINOCA, distinct mechanisms including Rho-kinase activation and TNF-α-driven α1-adrenergic upregulation drive vasospasm. Pericoronary FAI has demonstrated independent prognostic value across obstructive and non-obstructive coronary disease. Therapeutic strategies including sodium-glucose cotransporter-2 (SGLT-2) inhibitors, glucagon-like peptide-1 (GLP-1) receptor agonists, and peroxisome proliferator-activated receptor gamma (PPAR-γ) agonists show mechanistic promise for PVAT remodeling, though dedicated randomized evidence in non-diabetic INOCA populations remains limited. These findings support repositioning PVAT as a therapeutic target in non-obstructive ischemic disease, pending prospective validation.

## Introduction and background

Ischemia with non-obstructive coronary arteries (INOCA) is an umbrella term encompassing a range of pathophysiological processes, including coronary microvascular dysfunction (CMD), epicardial coronary spasm, and diffuse non-obstructive atherosclerosis. Myocardial infarction with non-obstructive coronary arteries (MINOCA) represents a related clinical entity characterized by acute myocardial infarction in the absence of obstructive coronary artery disease [1]. While INOCA refers to chronic or stable ischemic presentations in the absence of obstructive epicardial stenosis, MINOCA denotes an acute myocardial infarction phenotype sharing the same angiographic finding; the two entities differ importantly in their underlying mechanisms, clinical trajectories, and management strategies. In patients presenting with stable chest pain and referred for coronary angiography, 30% of men and 60% of women have non-obstructive disease, highlighting its epidemiological significance [2]. Despite its frequency, INOCA and MINOCA have long been under-recognized and under-treated, in part due to the lack of risk stratification and therapeutic interventions that are tailored to stenotic disease.

The pathophysiology of non-obstructive ischemic disease is multifactorial and remains incompletely understood. Coronary microvascular dysfunction, a condition in which the small resistance vessels of the heart fail to dilate adequately in response to increased myocardial demand, resulting in reduced coronary flow reserve (CFR) and elevated microvascular resistance, and producing ischemia despite the absence of obstructive epicardial stenosis, is considered a central mechanism in up to 40% of INOCA patients [3]. Emerging translational and clinical research has begun to reframe the adipose tissue surrounding coronary arteries, pericoronary perivascular adipose tissue (PVAT), a metabolically active fat depot that directly envelops the epicardial coronary vessels and communicates with the underlying vascular wall through paracrine and vasocrine signaling, but as an active paracrine and endocrine organ capable of profoundly modulating vascular tone and inflammation [4]. In healthy individuals, PVAT exerts anticontractile, anti-inflammatory, and anti-atherosclerotic effects through the constitutive secretion of adiponectin, a cardioprotective hormone produced by adipose tissue that promotes endothelial integrity, suppresses inflammation, and enhances insulin sensitivity, and nitric oxide (NO). PVAT contains at least three functionally distinct adipocyte populations, as identified by recent single-nuclear genome sequencing: uncoupling protein-1 (UCP-1)-expressing thermogenic adipocytes, AdipoQ-expressing endocrine adipocytes responsible for adiponectin secretion, and fibroinflammatory adipocytes that drive local vascular inflammation [5]. In the context of metabolic dysfunction, obesity, and insulin resistance, however, PVAT undergoes a deleterious phenotypic switch that suppresses adiponectin output and amplifies the release of pro-inflammatory mediators, creating a local coronary inflammatory milieu that may initiate or perpetuate microvascular dysfunction even in the absence of epicardial stenosis [6]. Notably, in the pre-clinical stage of INOCA, adiponectin levels may be transiently elevated as a compensatory response to early vascular stress, before progressive PVAT dysfunction leads to secretory failure [7]. This narrative review aims to critically appraise the mechanistic links between PVAT-derived adiponectin signaling failure and pro-inflammatory transformation in non-obstructive ischemic disease, to evaluate novel imaging strategies for PVAT assessment, and to identify promising endocrine-metabolic therapeutic targets that could rebalance the PVAT secretome to alleviate CMD and improve patient outcomes.

## Review

Methods

Search Strategy

A comprehensive literature search was conducted across PubMed/MEDLINE, Scopus, the Cochrane Library, and Google Scholar for studies. The search covered literature published from database inception to March 2025. Search strategies combined MeSH terms and free-text keywords using Boolean operators centered on perivascular adipose tissue (PVAT), adiponectin signaling, and non-obstructive ischemic heart disease (INOCA/MINOCA). Representative search terms included: ("perivascular adipose tissue" OR "PVAT") AND ("coronary microvascular dysfunction" OR "INOCA" OR "MINOCA"); ("adiponectin") AND ("endothelial dysfunction" OR "coronary artery disease"); and ("fat attenuation index" OR "FAI") AND ("pericoronary inflammation"). Supplementary targeted searches captured evidence on molecular pathways (AMP-activated protein kinase (AMPK), nuclear factor kappa B (NF-κB), endothelial nitric oxide synthase (eNOS), NOD-, LRR-, and pyrin domain-containing protein 3 (NLRP3)), imaging biomarkers (pericoronary fat attenuation index (FAI) on coronary computed tomography angiography (CCTA)), therapeutic interventions (sodium-glucose cotransporter-2 (SGLT2) inhibitors, glucagon-like peptide-1 (GLP-1) receptor agonists, peroxisome proliferator-activated receptor gamma (PPAR-γ) agonists), and sex-specific differences. Reference lists of eligible articles and clinical trial registries were additionally screened.

Inclusion Criteria

Eligible studies included original research (randomized controlled trials (RCTs) and observational studies), systematic reviews, meta-analyses, and mechanistic preclinical studies investigating PVAT biology, adiponectin pathways, coronary microvascular dysfunction, and related cardiometabolic conditions. Imaging studies reporting pericoronary FAI and studies evaluating relevant therapeutic interventions were also included. Only English-language, peer-reviewed full-text articles were considered.

Exclusion Criteria

Studies focusing solely on obstructive coronary artery disease, non-cardiovascular adipose tissue without mechanistic relevance, or lacking quantitative data were excluded. Editorials, case reports, duplicates, retracted articles, and methodologically flawed studies were similarly excluded.

Evidence Synthesis

Evidence was synthesized narratively, as the heterogeneity of included literature, spanning preclinical mechanistic studies, observational cohorts, imaging studies, and clinical trials, precluded quantitative pooling. Where pooled estimates from previously published meta-analyses are cited, they are clearly attributed to those source studies and reported with their original effect sizes and statistical parameters where available. The strength and directness of evidence is indicated throughout the review, distinguishing findings derived from clinical INOCA/MINOCA populations from those extrapolated from preclinical or general cardiometabolic studies.

Thematic Organization

Evidence was synthesized narratively across five domains: (1) PVAT physiology and the adiponectin-eNOS-NO anticontractile axis; (2) adiponectin signaling through adiponectin receptor 1 (AdipoR1)/adiponectin receptor 2 (AdipoR2), AMPK, and NF-κB; (3) PVAT phenotypic transition and coronary microvascular dysfunction in INOCA/MINOCA; (4) pericoronary FAI as a non-invasive imaging correlate of PVAT inflammation; and (5) endocrine-metabolic therapeutic targets with evidence for PVAT remodeling.

Biology and physiological function of perivascular adipose tissue

Perivascular adipose tissue is a distinct adipose depot surrounding most large- and medium-sized arteries, including the coronary arteries, but not intracranial vessels or pulmonary arteries [8]. Unlike traditional white or brown adipose tissue, PVAT is thermogenically intermediate, a mixed cell type containing mature adipocytes, macrophages, mast cells, lymphocytes, preadipocytes, fibroblasts, stem cells, and abundant extracellular matrix [9]. Recent single-nuclear genome sequencing has identified three functionally distinct adipocyte populations within PVAT: UCP-1-expressing thermogenic adipocytes, AdipoQ-expressing endocrine adipocytes responsible for adiponectin secretion, and fibroinflammatory adipocytes that drive local vascular inflammation [10]. In the coronary circulation, PVAT is contiguous with epicardial adipose tissue (EAT) through communicating vascular and lymphatic channels, and the distinction between the two depots becomes blurred. Its location adjacent to the adventitia facilitates paracrine communication independent of the systemic circulation, enabling bioactive molecules to penetrate the vessel wall via the vasa vasorum and directly influence vascular smooth muscle cells (VSMCs) and the coronary endothelium through an outside-in signaling axis that regulates coronary vasomotor function [11].

In lean, healthy individuals, PVAT functions as a paracrine vasorelaxant and anti-inflammatory organ, secreting perivascular relaxing factors (PVRFs) including adiponectin, hydrogen sulfide, nitric oxide, and angiotensin [11]. Adiponectin, secreted following β3-adrenoceptor activation by noradrenaline, activates AdipoR1 on endothelial cells, stimulating AMPK-dependent eNOS phosphorylation at Thr176, increasing bioavailable NO and inducing vasorelaxation [12]. Confirmed in human gluteal artery preparations, this effect is abolished by AdipoR1 blockade, establishing adiponectin as the dominant PVAT-derived vasoprotective mediator [13]. Beyond vasodilation, adiponectin downregulates vascular cell adhesion molecule-1 (VCAM-1) and E-selectin, suppresses NF-κB-mediated inflammatory gene transcription, inhibits macrophage-to-foam cell transformation, and limits VSMC proliferation and migration [14]. Adiponectin further exerts immunomodulatory effects by shifting macrophage polarization from M1 to M2, suppressing T-lymphocyte activation, and reducing dendritic cell-mediated inflammation [15]. It demonstrates antiapoptotic activity in endothelial cells and cardiomyocytes through AMPK-dependent caspase-3 inhibition and B-cell lymphoma 2 (Bcl-2) upregulation [16] and regulates mitochondrial autophagy via AMPK-mediated activation of Unc-51-like kinase 1 (ULK-1) and phosphoinositide 3-kinase (PI3K), preserving mitochondrial quality control and limiting cardiomyocyte injury under ischemic stress [17].

Adiponectin: structure, receptors, and signaling pathways

Adiponectin (AdipoQ, adipocyte complement-related protein of 30 kDa (ACRP30), gelatin-binding protein of 28 kDa (GBP-28)) is a 30-kDa collagen-domain-containing adipokine and the most abundant adipocyte-secreted protein, circulating at 5-30 μg/mL in healthy individuals [18]. Its encoding gene, *ADIPOQ*, is located on chromosome 3q27, a locus associated with susceptibility to type 2 diabetes and metabolic syndrome, with a plasma half-life of approximately 75-90 minutes for globular forms and longer for high-molecular-weight (HMW) multimers [19]. Adiponectin circulates as trimers (~67 kDa), hexamers (~140 kDa), and HMW multimers (>300 kDa), with the HMW form representing the metabolically dominant isoform in muscle and cardiovascular tissues. Endoplasmic reticulum assembly of these complexes is regulated by chaperones including endoplasmic reticulum oxidoreductin-1 alpha (Ero1-α), endoplasmic reticulum protein 44 (ERp44), disulfide-bond A oxidoreductase-like protein (DsbA-L), and glucose-regulated protein 94 (GRP94) [18].

Plasma adiponectin is paradoxically inversely correlated with visceral fat mass, insulin resistance, type 2 diabetes mellitus (T2DM), and cardiovascular risk, declining as adipose tissue expands [20]. This hypoadiponectinemia is driven by tumor necrosis factor-α (TNF-α)-mediated ADIPOQ transcriptional suppression, oxidative stress-impaired HMW assembly, and endoplasmic reticulum stress in hypertrophied adipocytes. Notably, in the pre-clinical stage of INOCA, adiponectin levels may be transiently elevated as a compensatory response to early vascular stress before progressive PVAT secretory failure ensues [21], potentially representing a therapeutic window for early intervention.

Adiponectin signals through AdipoR1, abundantly expressed in vascular endothelium and skeletal muscle, preferentially activating AMPK, and AdipoR2, which predominates in hepatocytes and activates PPAR-α. In cardiovascular tissues, T-cadherin serves as an auxiliary HMW adiponectin receptor implicated in intimal repair and plaque modulation [22]. The adaptor protein APPL1 bridges both receptors to downstream effectors including AMPK, mitogen-activated protein kinase (MAPK), Akt, and NF-κB [22]. Through AMPK activation, adiponectin phosphorylates eNOS at Ser1177, augmenting NO production and limiting reactive oxygen species via nicotinamide adenine dinucleotide phosphate, reduced form (NADPH) oxidase inhibition. Via NF-κB, adiponectin blocks inhibitor of nuclear factor kappa B (IκB) kinase activation, preventing p65 nuclear translocation and suppressing transcription of IL-6, IL-8, and monocyte chemoattractant protein-1 (MCP-1) [23].

PVAT dysfunction in non-obstructive ischemic disease: pathophysiological mechanisms

PVAT Dysfunction in INOCA: Mechanisms of Coronary Microvascular Dysfunction

Under conditions of insulin resistance, visceral adiposity, dyslipidemia, and oxidative stress, PVAT undergoes a dramatic phenotypic transition. The anti-inflammatory, anticontractile adipocyte phenotype is supplanted by a hypertrophic, hypoxic phenotype characterized by infiltration of M1-polarized macrophages, mast cells, and CD8+ T lymphocytes, with a corresponding collapse in adiponectin secretion and upregulation of a pro-inflammatory secretome [6,13]. This shift mirrors what occurs in visceral adipose tissue during metabolic syndrome but is of greater cardiovascular consequence due to PVAT's proximity to the coronary endothelium.

Key pro-inflammatory mediators released by dysfunctional PVAT include TNF-α, IL-6, IL-1β, leptin, resistin, MCP-1, and reactive oxygen species. These factors act in concert to promote endothelial dysfunction through several mechanisms: suppression of eNOS expression and activity; uncoupling of eNOS (leading to superoxide rather than NO production); upregulation of endothelin-1 and angiotensin II; increased expression of VCAM-1 and intercellular adhesion molecule-1 (ICAM-1); and promotion of leukocyte rolling and transmigration [9]. In addition to endothelial effects, pro-inflammatory PVAT adipokines promote VSMC hypertrophy, phenotypic switching from a contractile to a synthetic phenotype, and adventitial fibrosis, all of which increase coronary microvascular stiffness and reduce distensibility [8,24].

Loss of Adiponectin-eNOS Axis and Coronary Microvascular Dysfunction

The adiponectin-AMPK-eNOS signaling axis is the principal molecular circuit through which PVAT exerts vasoprotective effects on the coronary microvasculature. In PVAT from obese patients, adiponectin secretion is significantly reduced compared with lean individuals, and this reduction is directly correlated with loss of the anticontractile effect of PVAT in ex vivo preparations of human arteries [11,13]. Margaritis et al. demonstrated in human vessels that PVAT-derived adiponectin regulates eNOS phosphorylation at Ser1177 and Thr495, activating and inhibitory sites, respectively, and that adiponectin deficiency leads to eNOS uncoupling and increased superoxide production in coronary endothelial cells, effectively converting eNOS from a NO source to a source of oxidative stress [12].

In the context of INOCA, where CMD is the dominant mechanism, impaired endothelium-dependent vasodilation is a cardinal feature. CMD is operationally defined by a coronary flow reserve (CFR) below 2.5 and an index of microcirculatory resistance (IMR) above 25 U [25]. Hypoadiponectinemia has been independently associated with reduced CFR in non-diabetic, non-obese patients with stable angina and unobstructed coronaries, suggesting that PVAT-derived adiponectin deficiency may be mechanistically upstream of the microvascular dysfunction observed in INOCA [26].

PVAT Dysfunction in MINOCA: Mechanisms of Vasospasm and Acute Ischemia

In contrast to INOCA, where chronic microvascular dysfunction predominates, MINOCA, defined by acute myocardial infarction in the absence of obstructive (≥50%) coronary stenosis, involves distinct acute pathophysiological mechanisms including coronary vasospasm, plaque erosion, and microvascular embolism [27]. The following evidence pertains specifically to MINOCA and should not be extrapolated to INOCA without qualification.

Perivascular adipose tissue (PVAT) is increasingly recognized as an active paracrine organ that modulates coronary vascular tone through the release of adipokines, cytokines, and reactive oxygen species. Under physiological conditions, PVAT exerts anti-contractile and vasodilatory effects mediated by adiponectin and nitric oxide (NO); however, in pathological states, PVAT undergoes inflammatory remodeling, shifting toward a pro-inflammatory and vasoconstrictive phenotype [12].

Inflamed PVAT has been directly implicated in the pathogenesis of coronary vasospasm. A clinical study by Ohyama et al. demonstrated that patients with vasospastic angina exhibit significantly increased PVAT volume at spastic coronary segments, supporting a localized pathogenic role [28]. Complementing this, PET/CT-based imaging studies have shown that coronary vasospasm is associated with perivascular inflammation, with PVAT serving as a source of inflammatory mediators that influence adjacent vascular smooth muscle contractility [29]. Mechanistically, PVAT inflammation promotes coronary hyperreactivity via oxidative stress and Rho-kinase activation, leading to exaggerated vasoconstriction [30]. Notably, tumor necrosis factor-α (TNF-α) derived from inflamed PVAT has been shown to upregulate α1-adrenergic receptor expression on VSMCs, thereby amplifying vasoconstrictive responses [31].

Furthermore, imaging-based studies using pericoronary adipose tissue (PCAT) attenuation have demonstrated that increased coronary inflammation is associated with non-obstructive ischemic syndromes, including MINOCA, particularly in cases driven by vasospasm and microvascular dysfunction [32]. Notably, a clinical case report demonstrated that reduction in PVAT inflammation (measured by PCAT attenuation on CT) paralleled symptomatic improvement in a patient with MINOCA attributed to vasospastic angina, providing dynamic evidence of this relationship [33]. This finding, however, is derived from a single case report and should be regarded as hypothesis-generating rather than confirmatory evidence.

At the molecular level, dysfunctional PVAT exhibits upregulation of pro-inflammatory cytokines including IL-1β, IL-6, and MCP-1, which contribute to endothelial dysfunction, oxidative stress, and impaired vasodilatory capacity [31]. These processes create a feed-forward loop that enhances coronary smooth muscle contraction and reduces nitric oxide bioavailability, thereby predisposing to episodic vasospasm and ischemia in the absence of obstructive coronary disease.

Pericoronary fat attenuation index as a non-invasive biomarker of PVAT inflammation

The pericoronary fat attenuation index (FAI), derived from coronary computed tomography angiography (CCTA), though not yet fully validated for routine clinical use in INOCA, has emerged as a powerful non-invasive imaging biomarker of PVAT inflammation with direct applicability to non-obstructive coronary disease [34]. The FAI measures the average CT attenuation (in Hounsfield units, HU) of pericoronary adipose tissue within a standardized radial distance from the vessel wall. Inflamed PVAT, with its increased aqueous content, cellular infiltration, and lipid-poor, water-rich inflammatory exudate, displays higher (less negative) attenuation than quiescent fat, as the proportion of water relative to lipid increases with inflammation [6].

The landmark Cardiovascular RISk Prediction using Computed Tomography (CRISP-CT) study by Oikonomou and Antoniades, involving 3,912 patients followed for a median of 5.6 years, established that a pericoronary FAI ≥ -70.1 HU was an independent predictor of cardiac mortality and all-cause mortality beyond traditional risk factors, Framingham risk score, and even coronary plaque burden [35]. Critically, this prognostic signal was preserved in patients with non-obstructive coronary disease, affirming that pericoronary inflammation is a biologically active and clinically consequential process even without stenosis. A subsequent multicenter longitudinal cohort study of 450 CCTA-examined patients demonstrated robust associations between FAI scores and risks of cardiac mortality and major adverse cardiovascular events (MACE), with statistical significance maintained in the non-obstructive CAD subgroup [36].

In patients with MINOCA and Takotsubo syndrome, pericoronary fat attenuation index (pFAI) values were significantly elevated compared with controls, particularly during the acute phase, indicating the presence of transient coronary inflammation. Notably, pFAI values in MINOCA patients were higher within the first week of presentation but tended to normalize at later time points, suggesting a time-dependent resolution of inflammation [37]. A systematic review and meta-analysis including 20 studies and 7,797 patients demonstrated that FAI values are significantly higher in unstable compared with stable coronary plaques, with a mean difference of 4.50 Hounsfield units (95% CI: 1.10-7.89). Furthermore, elevated FAI was associated with a significantly increased risk of major adverse cardiovascular events (HR = 3.29, 95% CI: 1.88-5.76), supporting its role as a prognostic imaging marker [34].

New endocrine-metabolic therapeutic targets

SGLT-2 Inhibitors

The most compelling INOCA-specific evidence for SGLT-2 inhibitors comes from two phase II pilot trials by Tapp et al. and Quesada et al. [38,39]. In an all-female cohort of INOCA patients with CMD diagnosed by invasive thermodilution, excluding patients with diabetes or heart failure, empagliflozin 10 mg daily for 90 days significantly reduced circulating markers of endothelial dysfunction, including inflammatory endothelial microvesicles (eEVs) and reactive oxygen species, compared with no-CMD controls. The companion European Multicenter Adiponectin Biomarker Research Initiative (EMbArk) trial further demonstrated that sera from INOCA patients with CMD induced significantly higher coronary endothelial cell apoptosis and reduced viability compared to no-CMD controls, and that empagliflozin treatment significantly reversed these effects, reducing eEV release and eEV size, providing in vitro mechanistic evidence that SGLT2 inhibition directly attenuates the endothelial injury phenotype driven by INOCA-associated inflammation.

GLP-1 Receptor Agonists: Coronary Microvascular and PVAT Evidence

GLP-1 receptor agonists have demonstrated capacity to enhance coronary microvascular function through improved endothelial function, increased nitric oxide bioavailability, attenuation of oxidative stress, and reduced vascular inflammation, with evidence of improvement in myocardial blood flow and myocardial perfusion reserve, though inconsistencies remain across studies due to variability in patient populations and imaging methodologies, and dedicated INOCA-specific trials are currently lacking [40].

Mechanistically relevant to PVAT remodeling in non-obstructive ischemic disease, GLP-1 receptors are expressed in epicardial adipose tissue at substantially higher levels than in subcutaneous tissue, suggesting that GLP-1 rheumatoid arthritis (RAs) may directly target pericoronary adipose depots, the source of paracrine vascular inflammation in INOCA, for cardiovascular benefit beyond glycemic effects. Furthermore, a recent case report by Port et al. demonstrated that fat attenuation index (FAI) on CCTA identified coronary inflammation in a young woman with INOCA secondary to hypereosinophilic syndrome presenting with coronary vasospasm and non-obstructive coronary disease, representing the first reported use of FAI as a coronary inflammation biomarker in a non-atherosclerotic INOCA phenotype, establishing FAI as a relevant endpoint for future GLP-1 RA trials in INOCA [41]. Through activation of the cyclic adenosine monophosphate-protein kinase A (cAMP-PKA) pathway, GLP-1 RAs also enhance eNOS phosphorylation at Ser1177 independently of adiponectin, providing a complementary route to restored endothelium-dependent vasodilation in CMD [42]. Dedicated RCTs evaluating GLP-1 RAs specifically in non-diabetic INOCA patients with PVAT dysfunction are currently absent.

PPAR-γ Agonists: Adiponectin Upregulation in Non-Diabetic Coronary Disease

The most directly relevant clinical evidence for PPAR-γ agonism in non-diabetic coronary endothelial dysfunction comes from Pérez-Martínez et al., who demonstrated in a prospective interventional study that pioglitazone 30 mg daily for 12 weeks in 17 non-diabetic patients with stable coronary artery disease increased plasma adiponectin levels from a mean of 10.6 to 21.1 μg/mL (p=0.001) and significantly improved endothelium-dependent flow-mediated vasodilation (ED-FMD) from 4.45% to 8.43% (p=0.001), with effects reversing after a 12-week washout, providing direct evidence that PPAR-γ-mediated adiponectin upregulation restores endothelial function in non-diabetic patients with non-obstructive coronary pathology [19]. While concerns about fluid retention and bone fractures limit thiazolidinedione (TZD) use in some populations, selective PPAR-γ modulators and AdipoRon, a small-molecule AdipoR1/AdipoR2 agonist, represent next-generation approaches that bypass upstream metabolic barriers to adiponectin availability.

Anti-Inflammatory Strategies: Targeting Pericoronary PVAT Via FAI

Directly targeting pericoronary PVAT inflammation as measured by FAI, the Coat Protein Complex II (COPII) trial is the first prospective RCT to evaluate colchicine specifically for PVAT FAI reduction [43]. Forty patients with documented FAI ≥ -70.1 HU in the right coronary artery and/or LAD were randomized to colchicine 0.5 mg/day for 12 months, with FAI reduction as the primary endpoint, evaluating for the first time whether pharmacological suppression of NLRP3 inflammasome-driven PVAT inflammation translates into measurable pericoronary FAI reduction. COPII outcome data have not yet been published in peer-reviewed form; this evidence should therefore be regarded as hypothesis-confirming pending full publication.

Future Research Directions and Clinical Implications

Establishing whether PVAT-specific adiponectin deficiency causally drives CMD in INOCA, particularly in non-obese patients where local adipokine dysregulation may operate independently of systemic metabolic disease, remains a fundamental research priority. Prospective studies correlating PVAT adiponectin expression with invasive CFR and IMR in phenotyped INOCA cohorts are needed to confirm this mechanistic link. The three functionally distinct PVAT adipocyte populations identified by single-nuclear genome sequencing, thermogenic, endocrine, and fibroinflammatory, present tractable longitudinal targets, and serial FAI measurement alongside circulating HMW adiponectin profiling could define the compensatory pre-clinical window that may represent the optimal timing for early therapeutic intervention. FAI requires prospective validation as a treatment-stratification and therapeutic-response biomarker, with standardized acquisition protocols and clinically actionable thresholds, before it can transition into routine clinical practice; the pending COPII trial results represent an important first step in this direction.

On the therapeutic front, the phase II empagliflozin pilot data justify adequately powered, placebo-controlled RCTs of SGLT-2 inhibitors in non-diabetic INOCA patients with invasively confirmed CMD, incorporating FAI as a co-primary imaging endpoint alongside CFR and IMR. Dedicated GLP-1 receptor agonist trials in this population are similarly absent despite strong mechanistic rationale. Selective PPAR-γ modulators and AdipoRon represent next-generation approaches targeting adiponectin receptor signaling downstream of PVAT dysfunction, while localized coronary delivery strategies and combination regimens merit systematic evaluation. Critically, given the female predominance in INOCA and documented sex differences in PVAT biology, all future trials must prospectively incorporate sex-stratified analyses and ensure adequate representation of women to generate evidence with meaningful generalizability to the population most affected by this condition.

Limitations

Several limitations must be acknowledged. As a narrative review, the synthesis is susceptible to selection bias, with no formal assessment of publication bias or methodological quality across studies. The majority of mechanistic evidence derives from preclinical models and general cardiometabolic populations rather than rigorously phenotyped INOCA or MINOCA cohorts, limiting direct pathophysiological extrapolation. Therapeutic evidence for SGLT-2 inhibitors, GLP-1 receptor agonists, and PPAR-γ agonists is largely drawn from diabetic or obese populations, with dedicated randomized controlled trials in non-diabetic INOCA patients with invasively confirmed CMD either absent or restricted to small phase II pilot studies. FAI has not been prospectively validated as a treatment-stratification or therapeutic-response biomarker in INOCA or MINOCA, and standardized acquisition protocols and actionable thresholds remain undefined. Direct measurements of PVAT-specific adiponectin expression correlated with invasive microvascular indices, CFR and IMR, are lacking in this population, meaning the proposed mechanistic pathway, while biologically plausible, remains unconfirmed in clinical INOCA. Finally, sex-based differences in PVAT biology and adiponectin physiology are insufficiently captured across the included literature, limiting generalizability to women, who constitute the majority of INOCA patients. These limitations collectively underscore the need for prospective, sex-stratified, mechanistically anchored clinical trials integrating PVAT imaging biomarkers with invasive coronary physiological endpoints.

## Conclusions

Pericoronary PVAT dysfunction, through adiponectin signaling failure and pro-inflammatory phenotypic transition, represents an emerging and therapeutically actionable mechanism underlying coronary microvascular dysfunction and vasomotor dysregulation in non-obstructive ischemic heart disease. Pericoronary FAI offers a promising non-invasive window into this pathology, while endocrine-metabolic interventions, including SGLT-2 inhibitors, GLP-1 receptor agonists, and PPAR-γ agonists, hold mechanistic promise for restoring PVAT homeostasis. Prospective randomized trials integrating PVAT imaging biomarkers, invasive microvascular endpoints, and sex-stratified analyses are now needed to translate this biological framework into clinical practice and deliver targeted therapeutic options to a population that has long remained underserved.
